# Effects of genetics and sex on adolescent behaviors following neonatal ethanol exposure in BXD recombinant inbred strains

**DOI:** 10.3389/fnins.2023.1197292

**Published:** 2023-07-26

**Authors:** Jessica A. Baker, Megan K. Mulligan, Kristin M. Hamre

**Affiliations:** ^1^Department of Anatomy and Neurobiology, University of Tennessee Health Science Center, Memphis, TN, United States; ^2^Center for Behavioral Teratology, San Diego State University, San Diego, CA, United States; ^3^Department of Genetics, Genomics, and Informatics, University of Tennessee Health Science Center, Memphis, TN, United States

**Keywords:** alcohol, genetics, hippocampus, behavior, FASD, BXD RI strains

## Abstract

**Introduction:**

Fetal alcohol spectrum disorders (FASD) are the leading preventable neurodevelopmental disorders and two hallmark symptoms of FASD are abnormal behavior, and cognitive and learning deficits. The severity of alcohol’s teratogenic effects on the developing brain is influenced by genetics and sex. We previously identified recombinant inbred BXD mouse strains that show differential vulnerability to ethanol-induced cell death in the developing hippocampus, a brain region important in learning and memory. The present study aimed to test the hypothesis that strains with increased vulnerability to ethanol-induced cell death in the hippocampus have concomitant deficits in multiple hippocampal-related behaviors during adolescence.

**Methods:**

The current study evaluated the effects of developmental ethanol exposure on adolescent behavior in two BXD strains that show high cell death (BXD48a, BXD100), two that show low cell death (BXD60, BXD71), and the two parental strains (C57BL/6 J (B6), DBA/2 J (D2)). On postnatal day 7, male and female neonatal pups were treated with ethanol (5.0 g/kg) or saline given in two equal doses 2 h apart. Adolescent behavior was assessed across multiple behavioral paradigms including the elevated plus maze, open field, Y-maze, and T-maze.

**Results:**

Our results demonstrate that the effects of developmental ethanol exposure on adolescent behavioral responses are highly dependent on strain. The low cell death strains, BXD60 and BXD71, showed minimal effect of ethanol exposure on all behavioral measures but did present sex differences. The parental –B6 and D2–strains and high cell death strains, BXD48a and BXD100, showed ethanol-induced effects on activity-related or anxiety-like behaviors. Interestingly, the high cell death strains were the only strains that showed a significant effect of postnatal ethanol exposure on hippocampal-dependent spatial learning and memory behaviors.

**Discussion:**

Overall, we identified effects of ethanol exposure, strain, and/or sex on multiple behavioral measures. Interestingly, the strains that showed the most effects of postnatal ethanol exposure on adolescent behavior were the BXD strains that show high ethanol-induced cell death in the neonatal hippocampus, consistent with our hypothesis. Additionally, we found evidence for interactions among strain and sex, demonstrating that these factors have a complex effect on alcohol responses and that both are important considerations.

## 1. Introduction

Ethanol consumption during pregnancy can cause abnormal development and has been shown to be particularly detrimental to the developing brain (Moore et al., 2004; Riley and McGee, 2005; Guerri et al., 2009). Neurodevelopmental disorders associated with exposure to alcohol during prenatal development are referred to as fetal alcohol spectrum disorders (FASD) (Bertrand et al., 2005). In the United States, it is estimated that 2–5% of live births are adversely affected by prenatal alcohol exposure (May et al., 2014, 2018; Wozniak et al., 2019). Children with FASD exhibit long-lasting cognitive impairments such as deficits in learning and memory (Astley et al., 2009; Guerri et al., 2009; Mattson et al., 2011, 2019). Additionally, many individuals with FASD also exhibit other neurobehavioral symptoms such as hyperactivity, attention problems, and emotional dysregulation (Mattson and Riley, 2000; Riley and McGee, 2005; Hellemans et al., 2010; Mattson et al., 2011; Glass et al., 2014; Mattson et al., 2019). Interestingly, early twin studies established that an important factor in both the presence and severity of FASD is genetics, as a higher concordance of deficits are seen in human monozygotic twins compared to dizygotic twins (Christoffel and Salafsky, 1975; Chasnoff, 1985). In addition, evidence shows that children exposed to approximately equivalent amounts of alcohol at similar developmental timepoints, show variations in the severity of alcohol-induced deficits (Astley, 2010).

Similar cognitive impairments and behavioral abnormalities are seen in animal models of prenatal alcohol exposure and these models are a useful tool to investigate the underlying mechanisms behind alcohol-induced neurobehavioral alterations [as reviewed in (Chokroborty-Hoque et al., 2014; Patten et al., 2014; Fontaine et al., 2016)]. In addition, animal models are a useful tool for studying the role of genetics in the effects of alcohol exposure on the developing central nervous system. Numerous studies have examined differential vulnerability to ethanol’s teratogenic effects across differing genetic backgrounds (Goodlett et al., 1989; Ogawa et al., 2005; Green et al., 2007; Downing et al., 2009; Chen et al., 2011; Goldowitz et al., 2014; Lossie et al., 2014). Most studies examining multiple strains have focused on malformations and brain abnormalities in embryos or neonates while fewer studies have examined differential behavioral responses to developmental alcohol exposure. The handful of studies that have focused on differential behavioral responses to perinatal ethanol exposure have used either selectivity bred strains that show differential alcohol-related traits such as alcohol preference or typically only compared two or three strains (Gilliam et al., 1987; Riley et al., 1993; Thomas et al., 1998, 2000). These studies identified differential behavioral responses to perinatal ethanol exposure such as hyperactivity, deficits in motor coordination, and learning and memory deficits (Gilliam et al., 1987; Riley et al., 1993; Thomas et al., 1998, 2000). Another tool for studying genetic variation and differential behavioral responses is the BXD recombinant inbred (RI) strains of mice which have been generated by crossing C57BL/6 J (B6) and DBA/2 J (D2) strains and inbreeding progeny for over 20 generations (Taylor et al., 1999; Wang et al., 2016). The BXD strains differ in alcohol responses in adults and show differential vulnerabilities to several malformations and developmental abnormalities after exposure to alcohol during development (Crabbe and Belknap, 1992; Downing et al., 2012; Goldowitz et al., 2014; Cook et al., 2015; Baker et al., 2017; Théberge et al., 2019). However, the behavioral effects of developmental alcohol exposure have yet to be examined in the BXD RI panel.

Using one strain, a number of animal studies have examined the effects of developmental alcohol exposure on activity-related and anxiety-like behaviors. Many of these studies report hyperactivity and/or increased anxiety-like behaviors though these results can vary depending on species, level of alcohol exposure, and time of exposure (Dursun et al., 2006; Kim et al., 2013; Fish et al., 2016; Xu et al., 2018). These preclinical results are similar to clinical findings in children with FASD who can present internalizing behavior problems such as anxiety or mood disorders as well as attention deficit hyperactivity disorder (ADHD) (Khoury et al., 2018). Importantly, these symptoms are not just present in childhood but continue throughout adulthood (Coles et al., 2022).

The hippocampus is of particular interest as it plays a large role in many of the cognitive and behavioral abnormalities present in FASD, specifically impairments in learning, memory, and attention. In humans, hippocampal abnormalities and dysfunctions have been associated with impaired spatial working memory performance (Coles et al., 1991; Hamilton et al., 2003; Willoughby et al., 2008; Moore et al., 2021). Impaired hippocampal-dependent behaviors are also seen in animal models using several different behavioral paradigms [as reviewed in (Patten et al., 2014; Marquardt and Brigman, 2016)] that assessed multiple hippocampal-dependent behaviors such as spatial learning and memory (Kelly et al., 1988; Wozniak et al., 2004) and fear conditioning (Wagner and Hunt, 2006; Hunt et al., 2009; Brady et al., 2012; Hamilton et al., 2014). Deficits in working memory have been assessed using the standard Y-Maze and impairments in spatial recognition memory have been examined using a modified Y-Maze (Sarnyai et al., 2000; Subbanna et al., 2013; Basavarajappa et al., 2014; Subbanna and Basavarajappa, 2014; Cantacorps et al., 2017). Importantly, ethanol-induced cell death during early postnatal development has been associated with memory impairments later in life (Wozniak et al., 2004; Subbanna et al., 2013; Subbanna and Basavarajappa, 2014). Moreover, the hippocampus, specifically the CA1 region has been linked to anxiety-like behavior (Cha et al., 2016; Jimenez et al., 2018; Ghasemi et al., 2022; Wang et al., 2022).

Interestingly, recent studies report sex-related differences in a number of behaviors including emotional regulation, hyperactivity, and cognition in individuals with FASD (Sayal et al., 2007; Herman et al., 2008; Flannigan et al., 2018) and animal models (Kelly et al., 1988; Wozniak et al., 2004; Hellemans et al., 2010; Fidalgo et al., 2017). Moreover, our previous analysis identified a strong effect of sex on changes in hippocampal gene expression following neonatal ethanol exposure (Baker et al., 2022). One advantage of using multiple strains is that we have the ability to investigate genotype-by-sex interactions and recent evidence suggests that this interaction is crucial in a number of neurological disorders (Plummer et al., 2013; Schaafsma and Pfaff, 2014; Zhao et al., 2020; Blokland et al., 2022) but has not been fully investigated in relation to FASD. Many studies using rodent models investigate the effects of ethanol exposure on brain development and administer the ethanol exposure during the early postnatal period (Hamilton et al., 2011; Risbud et al., 2022). For mice, PD 7 is the middle of the brain growth spurt, a time during which neurons are completing migration and differentiation, establishing connections through synaptogenesis, dendritic arborization is ongoing, and natural programmed cell death is occurring, and is part of the third trimester-equivalent in humans (Gil-Mohapel et al., 2010; Alfonso-Loeches and Guerri, 2011; Marquardt and Brigman, 2016).

In the present study, we examined the effects of developmental ethanol exposure on adolescent behavior using selected BXD RI strains to assess whether cell death and behavior alterations co-occur as discussed below. Strains were selected based on previous work which identified BXD strains that showed increased vulnerability to ethanol-induced cell death in the neonatal hippocampus after exposure to postnatal ethanol while other BXD strains were resistant to these effects (Goldowitz et al., 2014). In addition, these BXD strains were shown to have differential gene expression changes in the neonatal hippocampus after exposure to alcohol during development (Baker et al., 2022). Using the same postnatal ethanol exposure paradigm that identified the BXD strains, we aim to further investigate the long-term effects of developmental alcohol exposure on cognition and behavior in these selected strains. We hypothesize that BXD strains that show increased vulnerability to ethanol-induced cell death in the hippocampus have concomitant deficits in multiple behavioral domains. Adolescent animals exposed to postnatal ethanol (equivalent to the third trimester in humans) were tested across a battery of behavioral tests to examine the effects of developmental alcohol exposure on activity, anxiety-like behavior, working memory, and spatial recognition memory. Both males and females were examined to investigate possible genotype-by-sex interactions and address the effect of sex on these behavioral measures as sex-specific behavioral impairments have been found in both humans and animal models (Ieraci and Herrera, 2007; Sayal et al., 2007; Herman et al., 2008; Kelly et al., 2009; Hellemans et al., 2010; May et al., 2017; Woods et al., 2018; Xu et al., 2018).

## 2. Materials and methods

### 2.1. Subjects

Original breeders were obtained from Dr. Robert Williams at the University of Tennessee Health Science Center (UTHSC) and the Jackson Laboratory (Bar Harbor, ME). All treatments and experiments were approved by the Institutional Animal Care and Use Committee at UTHSC. The present study aims to better understand the long-term effects of postnatal ethanol exposure in strains that show differential vulnerability to ethanol-induced cell death in the hippocampus of male and female mice. To test this, mouse strains were examined including, C57BL/6 J (B6), DBA/2 J (D2), and select BXD recombinant inbred (RI) strains that showed differential susceptibility to ethanol-induced cell death in the developing hippocampus (Goldowitz et al., 2014). BXD48a (previously named BXD96) and BXD100 showed higher susceptibility to ethanol-induced cell death in the hippocampus while BXD60 and BXD71 showed lower vulnerability.

Once all strains were acquired, breeding was conducted at UTHSC. Breeders were the products of on-site mating and thus breeders were not affected by excess stressors such as travel and relocation. Mice were maintained on a 12:12 hour light:dark cycle and given food and water *ad libitum*. Environmental enrichments (igloo house and Nestlets) were placed in each mouse cage throughout all experiments. Breeding cages were maintained with multiple male and female mice over 60 days of age. Breeders were checked several times per week and when female mice appeared pregnant, they were placed alone in a clean cage and monitored daily for pups. Pregnant dams were separated to (1) acclimate the dam to a new cage and reduce stress (2) control for differences in pup rearing with other adult male and female mice in original breeding cages and (3) to allow for close monitoring of pups without disturbing other breeders. On average dams were placed in a cage alone 1 week prior to birth. The date of birth was defined as postnatal day 0 (PD 0). In order to control for potential differences in maternal care, the first litter from each mother was not used for experiments. Only litters of 4 or more were kept while large litters were culled to 8 pups. Half of the pups in each litter were assigned to the ethanol groups and the other half the control group. If a litter contained more than one animal per treatment group, per sex, the litter mean for each treatment group and sex were calculated and used for statistical analysis.

### 2.2. Ethanol exposure

Neonatal mice were treated on postnatal day (PD) 7 which is a developmental time point during the third trimester-equivalent in humans. For mice, PD 7 is the middle of the brain growth spurt, a time during which neurons are completing migration and differentiation, establishing connections through synaptogenesis, dendritic arborization is ongoing programmed cell death is occurring (Gil-Mohapel et al., 2010; Alfonso-Loeches and Guerri, 2011; Marquardt and Brigman, 2016). For treatment, pups were brought to a separate testing room in their cage with their mother between 9:00 AM and 10:00 AM. Pups were placed in a clean cage on a heating pad while they were weighed, dosed, and then promptly placed back in their home cage with their mother. Litters were split with half of the pups in the ethanol group and half in the control group. As in previous studies (Goldowitz et al., 2014), ethanol treated animals received 20% ethanol in sterile saline though subcutaneous injection. The total dose of ethanol was 5.0 g/kg split in two 2.5 g/kg doses, given 2 h apart while controls received an isovolumetric volume of sterile saline (Goldowitz et al., 2014). This ethanol exposure represents an acute neonatal binge which has been shown to produce BACs of approximately 350 mg/dL in P7 neonatal mice (Goldowitz et al., 2014; Schaffner et al., 2020). Early prenatal and postnatal rodent studies of blood alcohol concentrations found no differences in BAC levels across multiple strains including B6 and D2 mouse strains (Goodlett et al., 1989; Boehm II et al., 1997). At the time of the first injection, pups were toe clipped for identification purposes during early development. On postnatal day 28, animals were weaned, separated by sex, and ear punched for easier identification purposes, as toe clip reading prior to behavioral testing can be an additional stressor (Castelhano-Carlos et al., 2010; Schaefer et al., 2010; Paluch et al., 2014).

### 2.3. Behavioral testing procedure and schedule

**Figure 1:**
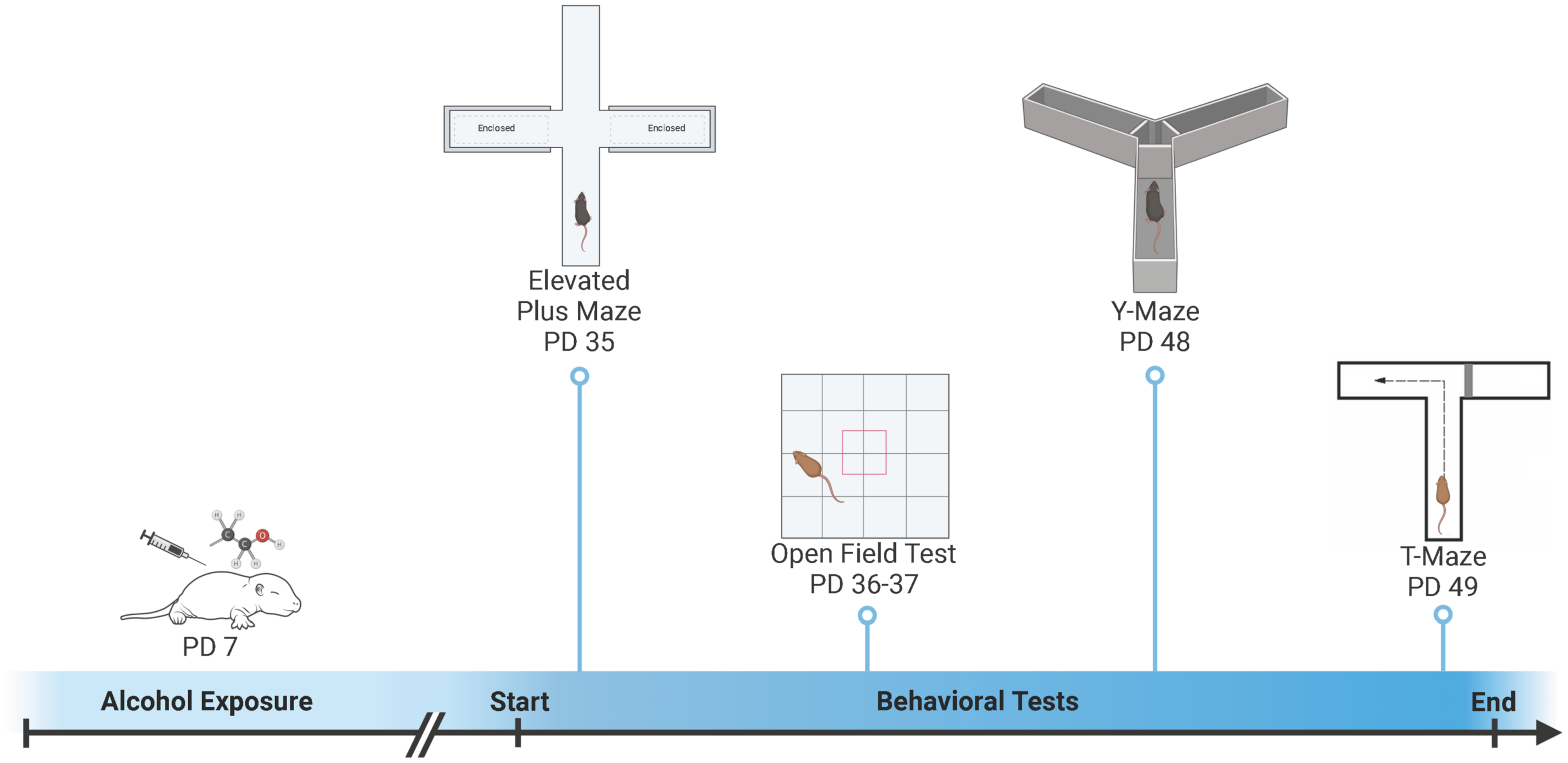
Overview of developmental ethanol exposure and behavioral testing. On postnatal day (PD) 7, pups were exposed to ethanol or saline vehicle via subcutaneous injection. Pups were then left undisturbed until behavioral testing. Activity-related and anxiety-like behaviors were measured during early adolescence (PD 35 – PD 37) using an Elevated Plus Maze (EPM) and Open Field (OF). Spatial learning and memory were measured during late adolescence (PD 48 – PD 49) using a Y-Maze and T-Maze. Created with BioRender.com.

The elevated plus maze (EPM) and open field (OF) were used to examine activity and anxiety-like behaviors and the Y-Maze and T-Maze were used to examine spatial learning and memory (Figure 1). All mice were examined in all behaviors (Fish et al., 2016; Fidalgo et al., 2017), however, some subjects were excluded from analysis due to technical issues (see Supplemental Table S1). The EPM and OF were conducted during early adolescence (EPM: PD 35.8 ± 1.1; OF Day 1: PD 36.8 ± 1.1; OF Day 2: PD 37.8 ± 1.1). Approximately, two-weeks later animals were tested in the Y-Maze and T-Maze during late adolescence (Y-Maze: PD 49.1 ± 1.3; T-Maze: PD 50.1 ± 1.3). The testing order was designed to test anxiety first using the elevated plus maze and open field, as anxiety is the most likely to be impacted by prior experience followed by the learning and memory tasks (i.e., Y-Maze and T-Maze) (Thomas et al., 2010; Risbud et al., 2022). In addition, we chose the learning tasks of the Y-Maze and T-Maze, that were different shapes and sizes in order to minimize carry-over effects.

All behavioral testing was performed in the Behavioral Core of the Neuroscience Institute at the University of Tennessee Health Science Center. Animals were acclimated for 1 h prior to testing which began between 10:00 AM −11:00 AM with noise level and lighting tightly controlled. Animals were placed in clean cages after testing and each apparatus was cleaned with 70% ethanol.

All behavior was tracked and recorded using ANY-maze Software version 4.99z (Stoelting Co., Wood Dale Illinois, and United States). For all behaviors, the number of entries is defined as 85% of the animal’s body, i.e., all four paws, to enter the zone, while their exit out of a zone requires 70% of the animal’s body to leave the zone (Any-maze Manual, Stoelting). If a litter contained more than one animal per treatment group, per sex, the litter mean for each treatment group and sex were calculated and used for statistical analysis. A minimum of 7 animals (from a minimum of 7 litters) per strain, per treatment group, per sex was used. The complete summary of animal numbers by strain, treatment, and sex are recorded in Supplementary Table S1.

#### 2.3.1. Elevated plus maze

The elevated plus maze (EPM) was used to examine anxiety-like behavior and locomotor activity as previously described (Bailey and Crawley, 2009; Fish et al., 2016; Xu et al., 2018). Mice were placed near the center of an EPM which is a plus-shaped (+) maze consisting of four arms (30 cm X 6 cm), two of which are open and two of which are enclosed with clear 15 cm walls. The runway was elevated 84 cm from the floor. Animals were tested for 5 min. The purpose of this test is to measure anxiety-like behavior and activity in mice. Therefore, total distance travelled in the maze and total number of line crossings as well as number of entries and percent time spent in each of the open and closed arms of the maze was determined.

#### 2.3.2. Open field

The open field (OF) was used to examine anxiety-like behavior and locomotor activity as previously described (Ieraci and Herrera, 2007; Bailey and Crawley, 2009; Fish et al., 2016; Xu et al., 2018). Mice were placed in a clear OF (40 cm X 40 cm X 40 cm) and allowed to explore the arena for 15 min. Animals were tested twice in the OF, each session 24 h apart. For each session, analysis was conducted at the following time bins: 0 min to 5 min (Bin 1), 5 min to 10 min (Bin 2), 10 min to 15 min (Bin 3), and total 15 min (Total). Activity was examined in the whole maze area, in the center of the maze (286 cm^2^), and in the edge of the maze (800 cm^2^) as a measure of thigmotaxis (Bailey and Crawley, 2009). The following measures were recorded in the entire maze area, center of the maze or edge of the maze: total distance travelled, time spent, and number of entries were evaluated across all time bins on day 1 and day 2.

#### 2.3.3. Y-maze

The Y-Maze was used to examine hippocampal-dependent spatial working memory as previously described (Holcomb et al., 1998; Subbanna et al., 2013; Basavarajappa et al., 2014; Cantacorps et al., 2017). The Y-Maze consists of three enclosed arms (12 cm X 5 cm X 5 cm) in the shape of a Y. To orient the animal to the location of each arm, shapes of various colors were placed on the walls around the Y-Maze. Each mouse was placed in the entry arm and allowed to explore freely through the maze for an 8-min session. The sequence of arms entered was recorded to measure spontaneous alternations. Correct alternation was recorded as three consecutive choices of the three different arms. Spontaneous alternations are calculated by dividing the total number of alternations by the total number of choices minus 2 (Holcomb et al., 1998; Subbanna et al., 2014; Cantacorps et al., 2017). Total distance travelled in the Y-Maze was also examined. Additionally, distance travelled, number of entries, and time spent in each arm was also examined.

#### 2.3.4. T-maze

The T-Maze was used to examine spatial working memory as previously described (Subbanna et al., 2014; Shivakumar et al., 2020). Briefly, the T-Maze consisted of three arms, one entry arm (50 cm X 10 cm) and two top arms (28 cm X 10 cm). As before, various shapes were placed around the maze. Mice were placed in the entry arm and allowed to freely explore for an 8-min training session. During the training session, one of the top arms was blocked and the mouse was only able to assess one of the top arms. The location of the blocked arm was randomized. After a 3-h interval, short-term memory was assessed, during which both top arms of the T-Maze were accessible. Mice were placed into the entry arm and allowed to explore both top arms for 3 min. The animal’s ability to discriminate between the two top arms was measured by examining time in the novel (previously blocked armed) compared to total time between both the novel and familiar, previously opened arm. The discrimination ratio [novel arm/(novel arm + familiar arm)] was used to calculate the time spent between both arms and the number of entries into both arms. Other measures recorded during both the training and short-term memory sessions included: total distance travelled in the whole maze as well as number of entries and time spent in the entry arm and opened arm. Additionally, during the short-term memory session the following measures were recorded in the novel (previously blocked arm): number of entries, time spent, and latency to enter.

### 2.4. Behavioral analysis

All behavior was exported from ANY-maze and analyzed using the following packages in the R (version 4.1) software environment: plyr package (version 1.8.6), ggplot2 package (version 3.3.3) (Wickham, 2016), and effectsize package (version 0.4.5). The effect of strain, sex, treatment, strain x sex interaction, strain x treatment interaction, sex x treatment interaction, and strain x sex x treatment interaction were examined across the six strains (BXD48a, BXD60, BXD71, BXD100, B6, and D2), two sexes (male and females), and two treatments (controls and ethanol). ANOVAs were used to examine multiple measures in R using the following input: measure.model<− lm(data = Dat,measure~Strain*Sex*Treatment), anova(measure.model). The effect size was calculated using Omega Squared confidence intervals in R using the following input: omega_squared(measure.model, partial = TRUE, ci = 0.09) (Lakens, 2013). Further analysis within each strain was calculated by two-way ANOVAs for effects of sex, treatment, and sex x treatment interactions in GraphPad Prism 7 (GraphPad Software Inc., San Diego, California). Additional descriptive statistics can be found in Supplementary materials.

## 3. Results

### 3.1. Adolescent body weights

Body weight was measured after the animal completed the EPM and again after the Y-Maze in all strains and both males and females. Body weights after both the EPM and Y-Maze showed significant effects of strain [EPM: *F*_5,183_ = 15.96, *p* < 0.001, *ω*^2^ = 0.36, 90% CI (0.26, 0.43); Y-Maze: *F*_5,186_ = 24.22, *p* < 0.001 *ω*^2^ = 0.27, 90% CI (0.17, 0.34)] and sex [EPM: *F*_1,183_ = 74.98, *p* < 0.001 *ω*^2^ = 0.49, 90% CI (0.41, 0.56); Y-Maze: *F*_1,186_ = 1999.89, *p* < 0.001 ω^2^ = 0.26, 90% CI (0.18, 0.35)]. However, there was no significant difference in body weight between control and ethanol animals at either age.

### 3.2. Strain effects

There were robust effects of strain on almost every measure across all four behavioral tests, (Table 1). Activity-related behavior such as total distance travelled, showed significant effects of strain across the elevated plus maze, open field, Y-Maze, and T-Maze. Anxiety-related behaviors such as number of entries into the closed arms of the elevated plus maze and time in thigmotaxis during the open field also showed significant effects of strain. Similarly, learning and memory measures showed significant effects of strain such as spontaneous alternations in the Y-Maze and time in novel arm of the T-Maze during the short-term memory trial. Since there were such robust effects of strains across all behavioral tests, each strain was analyzed separately for effects of treatment, sex, or treatment by sex interactions. Therefore, we further analyzed the effects of developmental ethanol exposure and sex in each strain separately while paying attention to cell death status.

**Table 1:** Significant effects of strain on body weight and behavioral measures.

	*F*-value (Dfm, DFd)	value of *p*	*ω* ^2^	90% CI [,]
Body weight				
Weight at elevated plus maze	15.96 (5, 183)	0.001	0.36	[0.26, 0.43]
Weight at Y-maze	24.22 (5, 186)	0.001	0.27	[0.17, 0.34]
Elevated plus maze				
Total distance travelled	52.02 (5,195)	0.001	0.54	[0.46, 0.60]
Number of entries to the open arms	14.90 (5, 195)	0.001	0.24	[0.15, 0.31]
Distance travelled in the open arms	53.74 (5, 195)	0.001	0.55	[0.47, 0.60]
Number of entries to the closed arms	13.21 (5, 195)	0.001	0.22	[0.13, 0.29]
Distance travelled in the closed arms	27.21 (5, 195)	0.001	0.37	[0.28, 0.44]
Open field				
Day 1- Total distance travelled	18.23 (5, 189)	0.001	0.29	[0.19, 0.36]
Day 1- Time in center	8.34 (5, 189)	0.001	0.15	[0.06, 0.21]
Day 1-Time in edge	8.80 (5, 189)	0.001	0.16	[0.07, 0.22]
Day 1-Number of entries to center	10.48 (5, 189)	0.001	0.18	[0.09, 0.25]
Day 1-Number of entries to edge	9.94 (5, 189)	0.001	0.17	[0.08, 0.24]
Day 2-Total distance travelled	30.42 (5, 189)	0.001	0.41	[0.31, 0.48]
Day 2-Time in center	6.60 (5, 189)	0.001	0.12	[0.04, 0.17]
Day 2-Time in edge	8.50 (5, 189)	0.001	0.15	[0.06, 0.21]
Day 2-Number of entries to center	11.05 (5, 189)	0.001	0.19	[0.10, 0.26]
Day 2-Number of entries to edge	13.05 (5, 189)	0.001	0.22	[0.13, 0.29]
Y-maze				
Spontaneous alternations	13.01 (5, 189)	0.001	0.22	[0.12, 0.28]
Total distance travelled	43.01 (5, 189)	0.001	0.50	[0.41, 0.26]
T-maze				
Training trial-total distance travelled	36.31 (5, 191)	0.001	0.45	[0.36, 0.52]
Training trial-number of entries to open arm	29.78 (5, 191)	0.001	0.40	[0.31, 0.74]
Training trial-latency to enter open arm	3.41 (5, 191)	0.01	0.05	[0.00, 0.09]
Short-term memory trial-total distance travelled	12.70 (5, 191)	0.001	0.21	[0.12, 0.28]
Short-term memory trial-number of entries to familiar arm	7.70 (5, 191)	0.001	0.13	[0.05, 0.20]
Short-term memory trial-time in familiar arm	4.10 (5, 191)	0.001	0.04	[0.01, 0.11]
Short-term memory trial-number of entries to novel arm	11.21 (5, 191)	0.001	0.19	[0.10, 0.26]
Short-term memory trial-time in novel arm	3.78 (5, 191)	0.001	0.06	[0.00, 0.10]
Short-term memory trial-latency to enter novel arm	2.33 (5, 191)	0.05	0.03	[0.00, 0.06]
Short-term memory trial-discrimination ratio-time	2.73 (5, 191)	0.05	0.04	[0.00, 0.07]

### 3.3. Moderate cell death strains: B6 and D2

**Figure 2:**
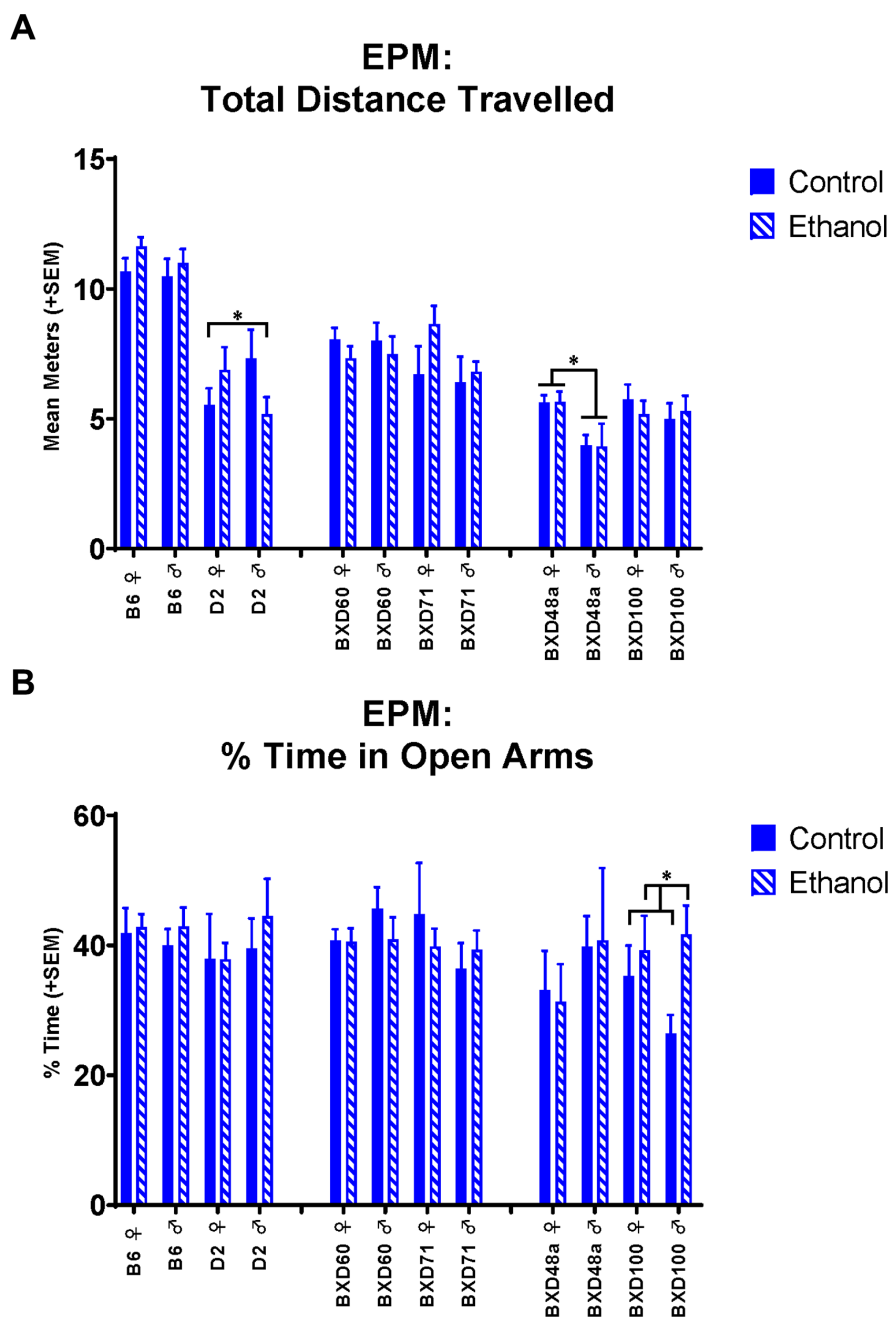
Effects of strain, ethanol, and sex on activity-related and anxiety-like behaviors in the Elevated Plus Maze (EPM). **(A)** There was a significant effect of strain (*p* < 0.001) on activity-related behaviors as measured by total distance travelled in the EPM. The D2 parental strain showed a significant ethanol-by-sex interaction (*p < 0.05; D2 ♀ Ethanol > D2 ♀ Control; D2 ♂ Ethanol < D2 ♂ Control). The high cell death strain, BXD48a, showed a significant effect of sex (*p < 0.01; BXD48a ♀ > BXD48a ♂). **(B)** The high cell death strain, BXD100, showed a significant effect of ethanol on anxiety-like behavior as measured by percent of time in the open arms of the EPM (*p < 0.05; BXD100 Ethanol > BXD100 Control). Striped bars represent animals exposed to postnatal ethanol while solid bars represent non-exposed control animals. Graphs are organized by parental strains (left), low cell death strains (middle), and high cell death strains (right). ♀, female, ♂, male.

For the EPM, there were no significant effects of ethanol exposure or sex for the B6 parental strain. In contrast, the D2 parental strain showed a significant interaction between ethanol exposure and sex for distance travelled [*F*_(1, 30)_ = 4.20, *p <* 0.05; Figure 2A] and number of line crossings [*F*_(1, 30)_ = 3.94, *p <* 0.05]. Ethanol exposure showed sex specific effects in activity-related measures in the D2 strain with increased distance travelled and number of line crossings in ethanol-exposed females compared to control females. In contrast, ethanol-exposed males showed decreased distance travelled and number of line crossings compared to control males.

**Figure 3:**
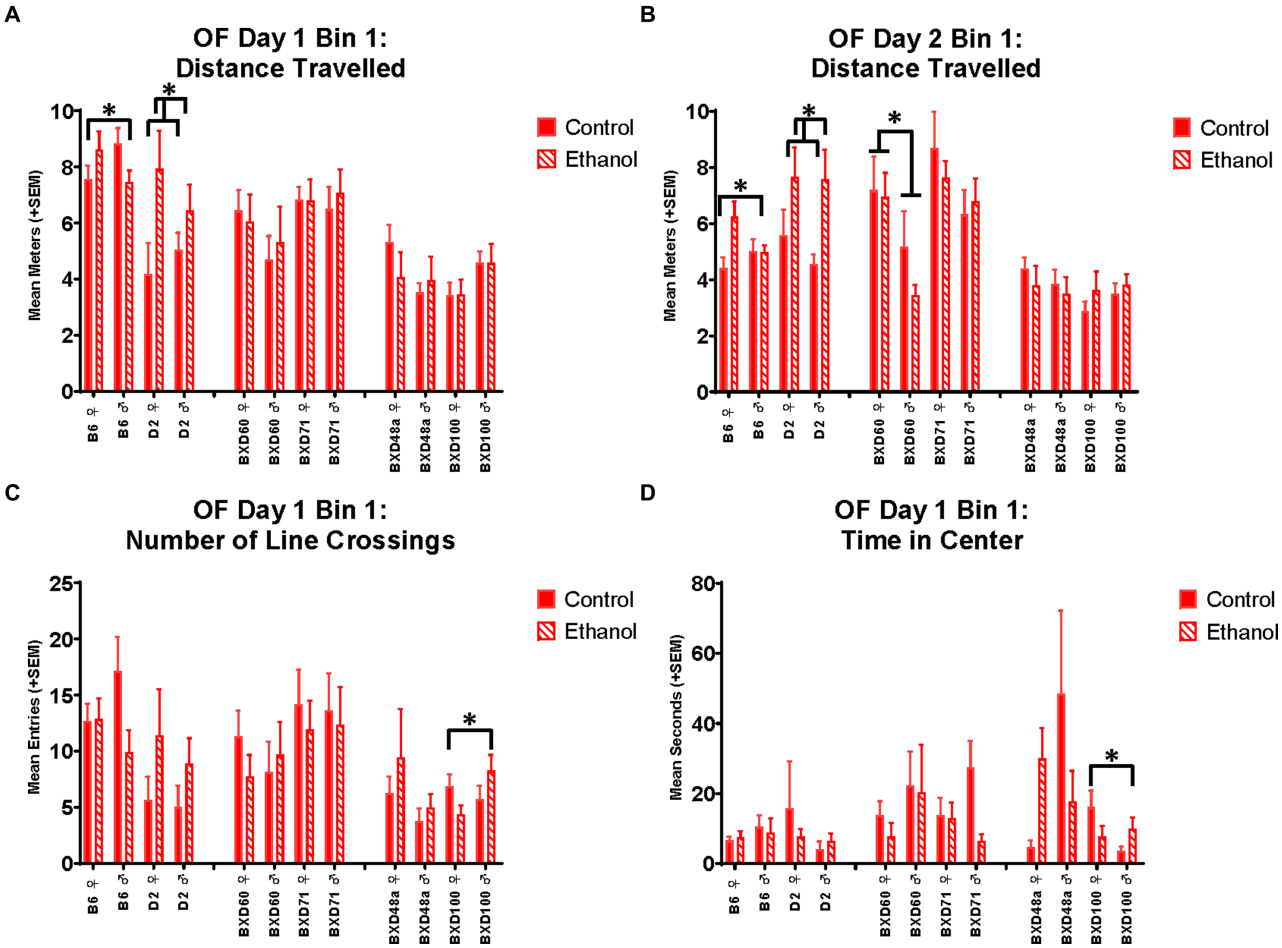
Effects of strain, ethanol, and sex on activity-related and anxiety-like behaviors during the first 5 min of the Open Field (OF). There was a significant effect of strain on distance travelled during the first 5 min (Bin 1) on **(A)** Day 1 (*p* < 0.001) and **(B)** Day 2 (*p* < 0.001). The parental B6 strain showed a significant ethanol-by-sex interaction (*p < 0.05; B6 ♀ Ethanol > B6 ♀ Control; B6 ♂ Ethanol < B6 ♂ Control) on distance travelled during the first 5 min. Additionally, the parental D2 strain showed a significant effect of ethanol (*p < 0.05; D2 Ethanol > D2 Control), regardless of sex, on Day 1 and Day 2. For the low cell death strain, BXD60, there was a significant effect of sex (*p < 0.01; BXD60 ♀ > BXD60 ♂) on distance travelled during the first 5 min of Day 2 but not Day 1. **(C)** There was also a significant effect of strain on number of line crossings during the first 5 min on Day 1 (*p* < 0.001) with the high cell death strain, BXD100 demonstrating a significant ethanol-by-sex interaction (*p < 0.05; BXD100 ♀ Ethanol < BXD100 ♀ Control; BXD100 ♂ Ethanol > BXD100 ♂ Control). **(D)** There were significant effects of strain (*p* < 0.01) on anxiety-like measures including time spent in the center of the Open Field on Day 1. For this measure, BXD100 strain showed similar ethanol-by-sex interactions (*p < 0.05; BXD100 ♀ Ethanol < BXD100 ♀ Control; BXD100 ♂ Ethanol > BXD100 ♂ Control). Striped bars represent animals exposed to postnatal ethanol while solid bars represent non-exposed control animals. Graphs are organized by parental strains (left), low cell death strains (middle), and high cell death strains (right). ♀, female, ♂, male.

In the OF, there was a significant interaction between ethanol exposure and sex in the B6 strains for distance travelled during the first 5 min on day 1 [*F*_(1, 41)_ = 5.24, *p* < 0.05; Figure 3A] and day 2 [*F*_(1, 41)_ = 4.70, *p* < 0.05; Figure 3B]. For both days, ethanol-exposed females showed increased distance travelled compared to control females. Ethanol-exposed males showed decreased distance travelled compared to control males on day one but there was no effect of ethanol-exposure in males on day 2. Non-sex-specific effects of ethanol exposure were present in the D2 strain with both males and females showing increased distance travelled during the first 5 min in the OF on day 1 [*F*_(1, 26)_ = 6.19, *p* < 0.05; Figure 3A] and day 2 [*F*_(1, 26)_ = 7.46, *p* < 0.05, Figure 3B].

Neither the B6 nor D2 strain showed any significant effects of ethanol-exposure or sex on spatial learning and memory measures in either the Y-Maze or the T-Maze.

### 3.4. Low cell death strains: BXD60 and BXD71

**Figure 4:**
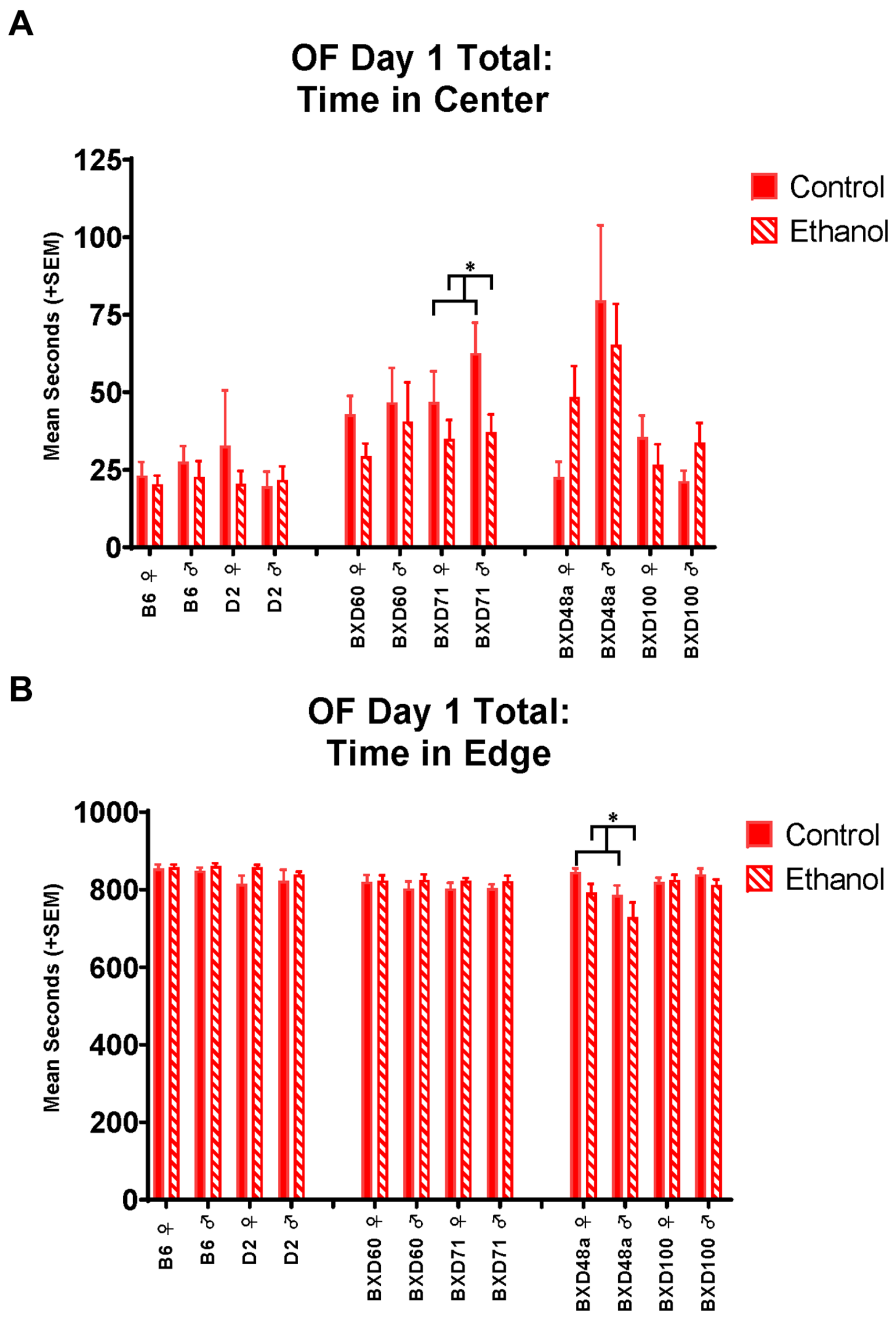
Effects of strain, ethanol, and sex on anxiety-like behaviors during the total 15 min of the Open Field (OF). **(A)** There was a significant effect of strain (*p* < 0.001) on time in center of the Open Field on Day 1. The low cell death strain, BXD71, showed a significant ethanol-by-sex interaction (*p < 0.05; BXD71 ♀ Ethanol < BXD71 ♀ Control; BXD71 ♂ Ethanol < BXD71 ♂ Control) in time in the center of the Open Field. **(B)** While there was not an overall effect of strain on time in edge of the Open Field on Day 1, there was a significant strain-by-ethanol interaction (*p* < 0.01). The high cell death strain, BXD48a, showed a significant effect of sex (*p* < 0.05; ♀ > BXD48a ♂) and ethanol (*p < 0.05; BXD48a Ethanol < BXD48a Control) on thigmotaxic behavior as measured by time in the edge of the Open Field. Striped bars represent animals exposed to postnatal ethanol while solid bars represent non-exposed control animals. Graphs are organized by parental strains (left), low cell death strains (middle), and high cell death strains (right). ♀, female, ♂, male.

The low cell death strains, BXD60 and BXD71, showed no significant effects of developmental ethanol exposure or sex activity-or anxiety-related measures in the EPM. In the OF, the BXD60 strain showed a significant effect of sex on distance travelled [*F*_(1, 28)_ = 7.85, *p* < 0.01; Figure 3B] and number of line crossings [*F*_(1, 28)_ = 4.93, *p* < 0.05] during the first 5 min that was only present on day 2 but not day 1. On day 2, BXD60 females showed greater levels of activity-related behaviors compared to BXD60 males, regardless of ethanol exposure. In contrast, the BXD71 strain showed no effect of sex or ethanol exposure on activity-related behaviors in the open field on day 1 or day 2; however, there was an effect of ethanol exposure on anxiety-related behaviors. Specifically, both male and female BXD71 animals exposed to ethanol during development spent significantly less time in the center of the OF on day 1 [*F*_(1, 31)_ = 5.14, *p* < 0.05; Figure 4A] with similar trends present on day 2 [*F*_(1, 31)_ = 3.32, *p* = 0.07].

In behavioral experiments related to spatial learning and memory, i.e., the Y-Maze and T-Maze, there were no effects of ethanol exposure or sex in the BXD60 strain. Similarly, the BXD71 strain showed no effects of ethanol or sex on learning and memory-related behavior. However, there was a significant effect of sex in the BXD71 strain on activity-related behaviors in both the Y-Maze and T-Maze (*p*’s < 0.05). BXD71 females showed greater distance travelled and number of line crossings compared to BXD71 males.

### 3.5. High cell death strains: BXD48a and BXD100

The high cell death strains, BXD48a and BXD100, showed effects of ethanol and/or sex in multiple behavioral measures. The high cell death strain, BXD48a, showed a significant effect of sex [*F*_(1, 29)_ = 8.74, *p* < 0.01] on activity-related measures in the EPM with females showing greater total distance travelled compared to males (Figure 2A). The BXD100 strain, showed a significant effect of ethanol exposure [*F*_(1, 34)_ = 4.78, *p* < 0.05] on anxiety-related measures in the EPM with ethanol-exposed subjects spending significantly more time in the open arms than the closed arms of the EPM compared to control subjects (Figure 2B).

On Day 1 of the OF, the BXD48a strain showed a significant effect of ethanol exposure [*F*_(1, 30)_ = 4.30, *p* < 0.05] and sex [*F*_(1, 30)_ = 5.28, *p* < 0.05] on thigmotaxis for the total 15 min. BXD48a females showed increased anxiety-like behavior with greater time in the edge compared to males (Figure 4B). However, ethanol-exposure decreased time spent in the edge in both sexes compared to controls (Figure 4B). On Day 2 of the OF, there was a significant treatment by sex interaction [*F*_(1, 30)_ = 4.51, *p* < 0.05] in activity-related behaviors where BXD48a males and females showed opposing effects of ethanol on number of line crossings during the whole 15 min. Ethanol-exposed males showed increased activity-like behavior compared to control males, while ethanol-exposed females showed decreased activity-related behavior compared to control females. Although not significant, there was a similar trend for ethanol by sex interaction [*F*_(1, 30)_ = 3.24, *p* = 0.08] for total distance travelled on Day 2 during the whole 15 min of the OF in the BXD48a strain.

For the BXD100 strain, there were significant treatment by sex interactions in both activity-related [*F*_(1, 33)_ = 4.83, *p* < 0.05] and anxiety-like [*F*_(1, 33)_ = 3.17, *p* < 0.05] behaviors during the first 5 min of the OF on Day 1. Similar to that seen in BXD48a, BXD100 males and females showed opposite effects of ethanol-exposure. BXD100 males exposed to ethanol during development showed increased number of line crossings (Figure 3C) and time spent in the center of the open field (Figure 3D) compared to control BXD100 males. In contrast, ethanol exposure decreased these behaviors in females. However, these ethanol and sex effects were only present in the first 5 min on Day 1 and not seen on Day 2 of the OF.

**Figure 5:**
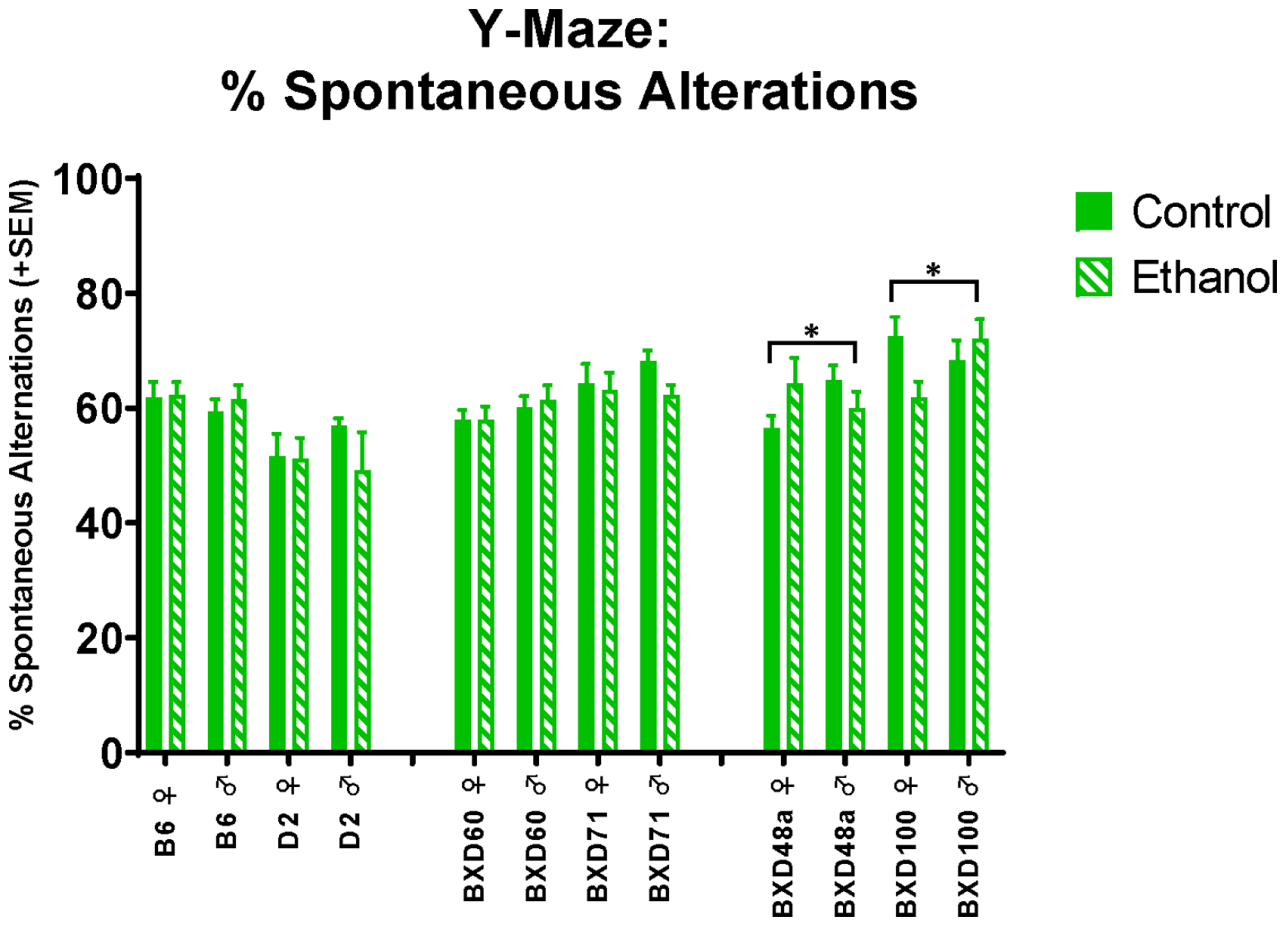
Effects of strain, ethanol, and sex spatial learning and memory in the Y-Maze. There was a significant effect of strain (*p* < 0.001) on percent of spontaneous alternations in the Y-Maze. Both high cell death strains, BXD48a and BXD100, showed significant ethanol-by-sex interactions, although effects were in opposing direction (*p < 0.05; BXD48a ♀ Ethanol > BXD48a ♀ Control; BXD48a ♂ Ethanol < BXD48a ♂ Control and *p < 0.05; BXD100 ♀ Ethanol < BXD100 ♀ Control; BXD100 ♂ Ethanol > BXD100 ♂ Control). Striped bars represent animals exposed to postnatal ethanol while solid bars represent non-exposed control animals. The graph is organized by parental strains (left), low cell death strains (middle), and high cell death strains (right). ♀, female, ♂, male.

Both high cell death strains showed significant interactions between ethanol exposure and sex on spatial learning and memory. There were significant ethanol by sex interactions in spontaneous alternations in the Y-Maze (Figure 5) for the BXD48a [*F*_(1, 29)_ = 3.96, *p* < 0.05] and BXD100 [*F*_(1, 34)_ = 4.75, *p* < 0.05] with males and females displaying opposite effects of ethanol exposure. In the BXD48a strain, males showed decreases in ethanol-induced spontaneous alternations while ethanol exposed females showed increases. However, the opposite was seen in BXD100, with ethanol exposure increasing spontaneous alternations in males and decreasing in females.

**Figure 6:**
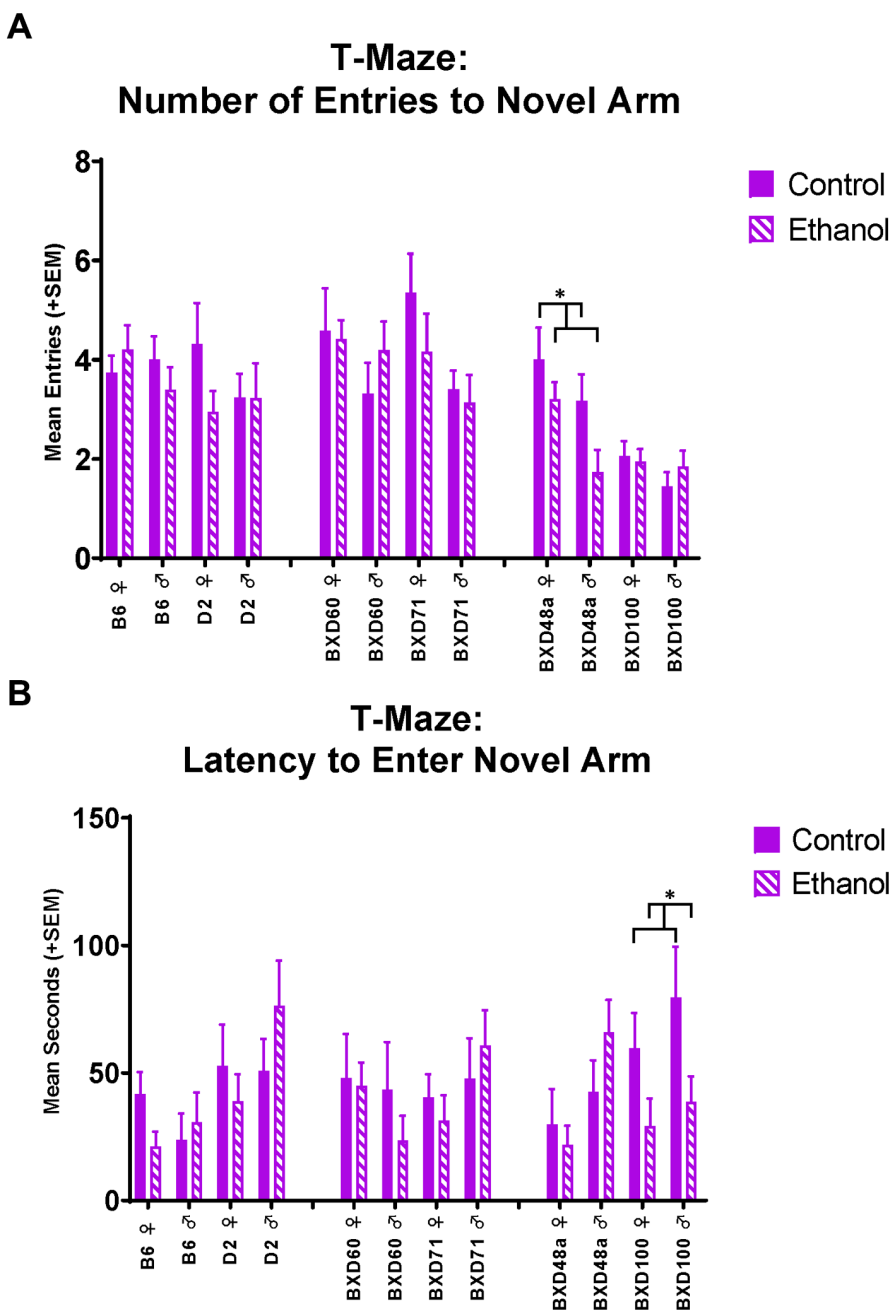
Effects of strain, ethanol, and sex on spatial learning and memory in the T-maze. **(A)** There was a significant effect of strain (*p* < 0.001) on number of entries to the novel, previously blocked, arm of the T-Maze during the short-term memory trial. The high cell death strain, BXD48a, showed a significant effect of ethanol in both sexes (*p < 0.05; BXD48a Ethanol < BXD48a Control) on number of entries to the novel arm. **(B)** In addition, there was also a significant effect of strain (*p* < 0.05) on latency to enter the novel arm of the T-Maze during the short-term memory trial. The other high cell death strain, BXD100, demonstrated a significant effect of ethanol (*p < 0.05; BXD100 Ethanol < BXD100 Control) on latency to enter the novel arm. Striped bars represent animals exposed to postnatal ethanol while solid bars represent non-exposed control animals. Graphs are organized by parental strains (left), low cell death strains (middle), and high cell death strains (right). ♀, Female, ♂, Male.

The high cell death strains also showed differences in spatial learning and memory measures in the T-Maze. During the short-term memory trial in the T-Maze, the BXD48a strain showed ethanol-induced impairments in learning and memory. Specifically, ethanol exposed subjects entered the novel arm of the maze significantly [*F*_(1, 28)_ = 4.89, *p* < 0.05; Figure 6A] less than control subjects. Importantly, there were no significant differences on entries into the familiar arm during the short-term memory trial. This is also seen by a trend (*p* < 0.1) in the discrimination ratio for number of line crossings during the short-term memory trial. In addition, there was also showed a significant effect of sex [*F*_(1, 28)_ = 4.89, *p* < 0.05] for number of entries into the novel arm of the maze with BXD48a males showing reduced entries overall compared to females. For the BXD100 strain, there was a significant effect of ethanol exposure [*F*_(1, 32)_ = 6.32, *p* < 0.05; Figure 6B] on latency to enter the novel arm during the short-term memory trial with ethanol-exposed subjects entering the novel arm sooner during the trial compared to controls.

## 4. Discussion

**Figure 7:**
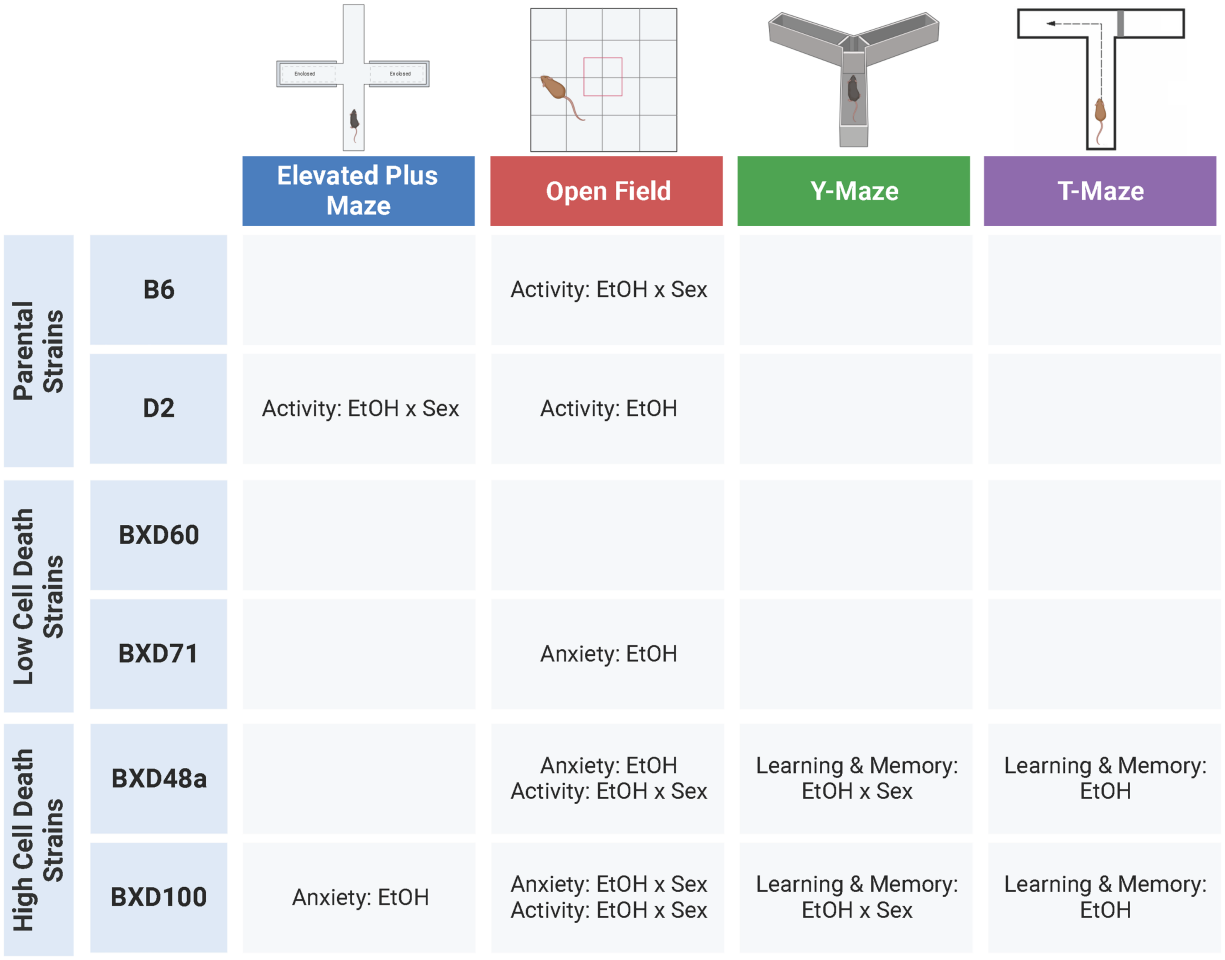
Summary of significant effects of ethanol across six strains and four behavioral tests. Adolescent behaviors were more effected by postnatal ethanol exposure in parental strains (B6, D2) and high cell death strains (BXD48a, BXD100). There were limited effects of ethanol exposure on adolescent behavior in the low cell death strains (BXD60, BXD71). Ethanol-induced alterations in learning and memory were only present in the high cell death strains. EtOH, Ethanol. Created with BioRender.com.

This study was designed to investigate the hypothesis that strains with relatively higher levels of cell death in the hippocampus after postnatal ethanol exposure will display alterations in behavioral responses in adolescence compared to low cell death strains, especially in tasks relevant to learning and memory that are reliant on the hippocampus (Baker et al., 2022). Previous work in adult animals has shown that there are baseline behavioral differences between strains using multiple behavioral paradigms (Chesler et al., 2005; Philip et al., 2010; Knoll et al., 2016; Neuner et al., 2016) and we replicate those findings by showing that there are significant strain difference in almost every behavioral measure in all four tests during adolescence. Furthermore, we show significant effects of sex for a number of behavioral measures among the strains. Although there were less effects of ethanol exposure compared to strain and sex effects, there were several treatment interactions between strain and/or sex in our behavioral measures, and several behaviors that showed ethanol-induced behavioral differences within specific strains. Interestingly, the strains that showed the most effects of postnatal ethanol exposure were the high cell death strains, BXD48a and BXD100 (Figure 7). In fact, these were the only strains to show effects of postnatal ethanol exposure on learning and memory tasks. Taken together these results emphasize the effects of genetics and sex on ethanol-induced behavioral alterations while at the same time underscoring the complex nature of these effects.

Developmental alcohol exposure has been shown to affect a wide-range of behavioral measures related to activity, emotional regulation, and learning and memory, though the presence or severity of these behavioral phenotypes vary depending on the level and timing of alcohol exposure as well as the age of behavioral testing [as reviewed in (Chokroborty-Hoque et al., 2014; Patten et al., 2014; Marquardt and Brigman, 2016)]. While molecular dysfunction and developmental abnormalities such as synaptic activity and apoptosis have been extensively studied in animals exposed to postnatal ethanol (equivalent to the third trimester in humans), behavioral responses to exposure at this developmental time point have been understudied in comparison. Many behavioral studies have examined the effects of chronic exposure to prenatal ethanol (Chokroborty-Hoque et al., 2014; Patten et al., 2014; Marquardt and Brigman, 2016) while fewer studies have examined behavioral effects to postnatal alcohol exposure (Marquardt and Brigman, 2016). In addition, many of the studies that do investigate the effects of postnatal ethanol exposure on behavioral responses use a chronic exposure paradigm across multiple postnatal days with fewer studies examining the behavioral effects of acute postnatal alcohol exposure. In our current model, we used an acute one-day ethanol exposure paradigm which could explain why we did not replicate some previous studies that examine the effects of ethanol treatment on adolescent behavior (Hunt et al., 2009; Patten et al., 2014; Marquardt and Brigman, 2016).

The overall statistical analysis comparing all strains, sexes, and treatment groups found little significant differences, however, further investigation within each strain revealed effects of treatment and/or sex by treatment interactions for many behavioral measures in almost all strains. We propose that the overwhelmingly large effects of strain, followed by sex in many measures may have obscured the effects of ethanol treatment in the overall analyses. This is not unexpected based on previous literature that has shown large strain effects in the BXD recombinant inbred strains in behavioral measures without drug exposure during adulthood (Chesler et al., 2005; Philip et al., 2010; Knoll et al., 2016; Neuner et al., 2016). We have expanded previous results by investigating the effects of sex and strain during adolescence. Moreover, this is the first study to evaluate the long-term effects of ethanol exposure during brain development on adolescent behaviors in our genetic reference population.

The BXD strains used in the current study were selected for their differential vulnerability to hippocampal cell death after exposure to postnatal ethanol (Goldowitz et al., 2014). The BXD100 and BXD48a strains were susceptible to high levels of ethanol induced cell death in the CA1 region of the hippocampus while the BXD60 and BXD71 strains were resistant to ethanol-induced cell death showing little to no difference compared to control animals (Goldowitz et al., 2014). This previous study also included the B6 and D2 parental strains which showed moderate levels of hippocampal cell death after postnatal ethanol exposure (Goldowitz et al., 2014).

While almost all strains showed effects of postnatal ethanol exposure in at least one measure of behavioral response, the strains that showed the most behavioral alterations after developmental ethanol exposure were the B6 and D2 parental strains as well as the high cell death strains, BXD100 and BXD48a (Figure 7). In these four strains, we observed many anxiety-like and/or activity-related behaviors that were significantly affected by postnatal ethanol exposure and in many of these measures there were sex-specific differences within the strain. The low cell death strains, BXD60 and BXD71, showed minimal effects of treatment in all behavioral tests. In the BXD60 strain, we did not observe effects of postnatal ethanol exposure on any of our behavioral measures. The BXD71 strain did show significant treatment effects in limited activity-related and anxiety-like behaviors.

The high cell death strains, BXD100 and BXD48a, were the only strains that showed significant effects of postnatal ethanol exposure in hippocampal-dependent spatial learning and memory assessments. The treatment effects in the BXD100 and BXD48a were often sex-specific and the direction of the behavioral response after postnatal ethanol exposure did not always indicate impairments in spatial learning. For example, spontaneous alternations in the Y-Maze showed significant treatment by sex interactions with ethanol-exposed BXD100 females and BXD48a males demonstrating impaired spatial memory. However, ethanol exposure had the opposite effects in the other sex of each strain: BXD100 male and BXD48a females. For the T-Maze, BXD100 ethanol-exposed males and females showed faster exploratory behavior in the novel arm during the short-term memory trial, indicative of enhanced short-term memory. In contrast, both male and female BXD48a mice exposed to postnatal ethanol showed reduced entries into the novel arm of the T-Maze indicative of impaired short-term memory. In addition, there was a trend toward significant effect of treatment for BXD48a ethanol-exposed discrimination between the arms of the T-Maze, indicating impaired short-term memory compared to non-exposed control BXD48a. While the high cell death strains did show effects of postnatal ethanol exposure on learning and memory behaviors, the relationship is more complex, not always indicating impairments. However, we want to emphasize that the only strains that showed effects in learning and memory tasks were the high cell death strains. More complex tasks that measure hippocampal related behaviors in a more in-depth manner and are less confounded by movement, may better clarify this in the future.

Our current study focused on examining strains that show differential cell death in the hippocampus after ethanol exposure during development as well as hippocampal-dependent learning and memory. However, the effects of ethanol-induced cell death during postnatal development in other brain regions can also affect long-term behavioral and cognitive measurements. For example, our previous study also identified differential susceptibility to ethanol-induced cell death in the cortex of BXD mice after postnatal alcohol exposure (Goldowitz et al., 2014). Interestingly, the level of cell death in each brain region showed uniformity in some strains while other strains showed regional specificity. For example, while the BXD100 strain was identified as a vulnerable strain for cell death in the hippocampus as well as the cortex, the BXD71 strain was resistant to cell death in the hippocampus but showed high susceptibility to cell death in layers 2/3 of the cortex (Goldowitz et al., 2014). Ethanol-induced cell death in other brain regions involved in cognition and attention, such as the cerebral cortex, could also impair learning and memory and may have had a strong impact on the results.

Previously, we examined gene expression differences in the neonatal hippocampus after acute ethanol exposure in the same mouse strains used in the current study (Baker et al., 2022). Similar to the present findings, we found sex-specific effects of ethanol within each strain although few effects of sex were consistent across all strains. In addition, we previously identified ethanol-induced gene expression changes related to learning and memory that were unique in the high cell death strains, BXD48a and BXD100, such as, *Gadd45g* [growth arrest and DNA damage inducible gamma (Sultan and Sweatt, 2013; Sepp et al., 2017)], *Elavl2* [ELAV like RNA binding protein (Ustaoglu et al., 2021)], *Igf2r* [insulin like growth factor 2 receptor (Beletskiy et al., 2021)], and *Vegfa* [vascular endothelial growth factor A (Ping et al., 2019)]. Future studies could further investigate the role of these genes on long-term behavioral alterations induced by early developmental alcohol exposure.

We observed significant effects of sex and/or interactions between sex and ethanol exposure in many behavioral analyses. Every one of the six strains examined showed an effect of sex or interaction of sex and treatment in at least one of our behavioral measures. Interestingly, more sex-specific behaviors were seen in the anxiety-like and activity-related behaviors. However, for any given behavioral measure the effects of sex were often strain-specific and not seen across all strains. Likewise, the direction of sex-specific differences was not consistent across all behavioral measures and/or strains. This suggests a complex relationship between developmental ethanol exposure, sex, and strain on adolescent behavior. It is important to note that the original paper that identified the BXD strains did not report the sex of the subjects examined for the CA1 hippocampal cell death analysis but did use both male and female subjects (Goldowitz et al., 2014). Moreover, age-related sex-specific differences in the hippocampus have been identified (Nunez and McCarthy, 2007). Interestingly in our previous gene expression study on these strains (Baker et al., 2022), we found significant effects of sex on ethanol-induced gene expression in the neonatal hippocampus within each strain. However, there were significant ethanol-induced gene expression changes across all strains and sex-dependent effects were only observed within a strain (Baker et al., 2022).

While we saw an effect of ethanol exposure within strains, our treatment effect on hippocampal learning and memory were not as robust as previous studies have found. While part of this could be strain-specific, we did not observe impaired spatial learning and memory in the B6 strains which is a highly used strain in behavioral studies assessing the effects of developmental alcohol exposure. An explanation for why we did not have a larger ethanol effect on behavioral responses could be due to the type of behavioral tests performed and the age of behavioral testing. For example, behavioral studies in adolescent animals exposed to acute postnatal ethanol have observed learning and memory impairments using more complex behavioral measures such as the Morris water maze, object recognition test, fear conditioning, and radial arm maze (Wozniak et al., 2004; Wagner and Hunt, 2006; Ieraci and Herrera, 2007; Wagner et al., 2014). Many of these experimental tests also included either a positive component such as a food pellet reward or a negative component such as a foot shock or forced water placement (Wozniak et al., 2004; Ieraci and Herrera, 2007; Wagner et al., 2014). In addition, all animals were tested in all behavioral tasks and while the order of the task was chosen to limit carry-over effects based on prior literature (Thomas et al., 2010; Risbud et al., 2022), this is still a limitation. Also, it is important to note that the behavioral tests used in the current study were highly dependent on activity. Since there were such robust effects of strain on activity levels, this could be overriding some of our ethanol-related effects. Future studies examining differential behavioral responses after exposure to developmental alcohol in these strains should take into account the significant strain effect on activity.

In addition, our behavioral results did show large individual variability within a strain, sex, and exposure group for many of our measures, especially in ethanol-exposed animals. This large variation could be due to the age of behavioral testing, as adolescent mice tend to show more behavioral variability than adult mice (Brust et al., 2015). Future studies could address large variation in behavioral measures by adding more subjects per group and running additional analyses to identify outliers. Although our study found large variability in animal behavior, we were still able to identify multiple behavioral measures effected by acute postnatal ethanol exposure including differences in activity, anxiety, and learning and memory behaviors in the BXD strains and B6 and D2 parental strains. Moreover, we assessed behavior on strains that showed differential cell death in the CA1 region of the hippocampus after ethanol exposure on postnatal day 7 and further studies can address cell counts in adult animals after behavioral testing.

This study emphasizes the importance of the role of genetics in ethanol-induced behavioral alterations. Moreover, the results are further influenced by sex as shown by strain-specific effects of sex. Our results support our hypothesis that adolescent behaviors were most affected by acute neonatal ethanol exposure in BXD strains that showed high ethanol-induced cell death in the postnatal hippocampus, particularly in behaviors related to learning and memory, which are highly dependent on the hippocampus. In contrast, BXD strains that showed low vulnerability to ethanol-induced cell death demonstrated limited effects of ethanol exposure on adolescent behavior. In conclusion, we found evidence for interactions among strain and sex, demonstrating that these factors have a complex effect on ethanol-induced responses and that both are important considerations for evaluating ethanol’s effects. These results support the inclusion of multiple strains and the evaluation of both males and females in behavioral studies examining the effects of developmental alcohol exposure. By evaluating multiple strains and both sexes, we can better understand the effects of genetic background and sex on alcohol-induced neurobehavioral abnormalities.

## Data availability statement

The raw data supporting the conclusions of this article will be made available by the authors, without undue reservation.

## Ethics statement

The animal study protocol was approved by the Institutional Animal Care and Use Committee of the University of Tennessee Health Science Center.

## Author contributions

JB, MM, and KH contributed to conception and design of the study. JB organized the database and performed the analyses under guidance of MM and KH. JB wrote the first draft of the manuscript with guidance from KH. All authors contributed to manuscript revisions, read, and approved the submitted version.

## Funding

This research was funded by the National Institute on Alcohol Abuse and Alcoholism R01AA023508 (KH), F31AA026498 (JB), T32AA013525–21 (JB) and University of Tennessee Health Science Center CORNET Award.

## Conflict of interest

The authors declare that the research was conducted in the absence of any commercial or financial relationships that could be construed as a potential conflict of interest.

## Publisher’s note

All claims expressed in this article are solely those of the authors and do not necessarily represent those of their affiliated organizations, or those of the publisher, the editors and the reviewers. Any product that may be evaluated in this article, or claim that may be made by its manufacturer, is not guaranteed or endorsed by the publisher.
