# Supplementary material for: Effects of genetics and sex on adolescent behaviors following neonatal ethanol exposure in BXD recombinant inbred strains
Source: Front Neurosci. 2023 Jul 26;17:1197292. doi: 10.3389/fnins.2023.1197292 (PMC10410115; doi:10.3389/fnins.2023.1197292)
Supplement: Supplementary file 1 [file Table_1.DOCX]

Supplementary Material

Effects of Genetics and Sex on Adolescent Behaviors Following Neonatal Ethanol Exposure in BXD Recombinant Inbred Strains

Jessica A. Baker, Megan K. Mulligan, Kristin M. Hamre*

*** Correspondence:** Corresponding Author: [khamre@sdsu.edu](mailto:khamre@sdsu.edu)

**Supplementary Table 1. Litter numbers used for behavioral experiments.** Only one animal per strain, per treatment, per sex, per litter was used. If a litter contain more than one animal per treatment group, per sex, the litter mean for each treatment group and sex were calculated and used for statistical analysis.

| **Strain** | **Sex** | **Treatment** | **EPM** | **OF** | **Y-Maze** | **T-Maze** |
| --- | --- | --- | --- | --- | --- | --- |
| B6 | Female | Control | 12 | 12 | 12 | 12 |
| B6 | Female | Ethanol | 12 | 12 | 12 | 12 |
| B6 | Male | Control | 11 | 10 | 11 | 11 |
| B6 | Male | Ethanol | 11 | 11 | 9 | 11 |
| BXD100 | Female | Control | 9 | 9 | 9 | 9 |
| BXD100 | Female | Ethanol | 9 | 9 | 9 | 9 |
| BXD100 | Male | Control | 10 | 9 | 10 | 10 |
| BXD100 | Male | Ethanol | 10 | 10 | 10 | 10 |
| BXD48a | Female | Control | 8 | 8 | 8 | 7 |
| BXD48a | Female | Ethanol | 8 | 8 | 8 | 8 |
| BXD48a | Male | Control | 8 | 9 | 8 | 9 |
| BXD48a | Male | Ethanol | 9 | 9 | 9 | 8 |
| BXD60 | Female | Control | 8 | 8 | 8 | 8 |
| BXD60 | Female | Ethanol | 8 | 8 | 7 | 8 |
| BXD60 | Male | Control | 8 | 8 | 8 | 8 |
| BXD60 | Male | Ethanol | 8 | 8 | 8 | 8 |
| BXD71 | Female | Control | 7 | 7 | 7 | 7 |
| BXD71 | Female | Ethanol | 8 | 8 | 6 | 8 |
| BXD71 | Male | Control | 10 | 10 | 10 | 10 |
| BXD71 | Male | Ethanol | 11 | 10 | 11 | 10 |
| D2 | Female | Control | 8 | 7 | 8 | 8 |
| D2 | Female | Ethanol | 8 | 7 | 7 | 7 |
| D2 | Male | Control | 9 | 7 | 9 | 9 |
| D2 | Male | Ethanol | 9 | 9 | 9 | 8 |
